# Nuclear gene mutations as the cause of mitochondrial complex III deficiency

**DOI:** 10.3389/fgene.2015.00134

**Published:** 2015-04-09

**Authors:** Erika Fernández-Vizarra, Massimo Zeviani

**Affiliations:** Mitochondrial Biology Unit, Medical Research CouncilCambridge, UK

**Keywords:** oxidative phosphorylation (OXPHOS), mitochondrial diseases, genetic mutations, complex III deficiency, complex III assembly

## Abstract

Complex III (CIII) deficiency is one of the least common oxidative phosphorylation defects associated to mitochondrial disease. CIII constitutes the center of the mitochondrial respiratory chain, as well as a crossroad for several other metabolic pathways. For more than 10 years, of all the potential candidate genes encoding structural subunits and assembly factors, only three were known to be associated to CIII defects in human pathology. Thus, leaving many of these cases unresolved. These first identified genes were *MT-CYB*, the only CIII subunit encoded in the mitochondrial DNA; *BCS1L*, encoding an assembly factor, and *UQCRB*, a nuclear-encoded structural subunit. Nowadays, thanks to the fast progress that has taken place in the last 3–4 years, pathological changes in seven more genes are known to be associated to these conditions. This review will focus on the strategies that have permitted the latest discovery of mutations in factors that are necessary for a correct CIII assembly and activity, in relation with their function. In addition, new data further establishing the molecular role of LYRM7/MZM1L as a chaperone involved in CIII biogenesis are provided.

## Introduction

Within the group of mitochondrial diseases or more specifically, OXPHOS disorders, isolated mitochondrial complex III (CIII) deficiencies are among the least frequently diagnosed. It is possible that these deficits are not rarer than those of the other complexes, but their diagnosis may be more difficult due to the lack of histological and biochemical hallmarks in skeletal muscle biopsies, e.g., no COX negative or ragged red fibers (DiMauro et al., 2013). Also, different protocols used in different labs to measure CIII enzymatic activity can also introduce some bias to detect defects (Medja et al., 2009). Typical to mitochondrial syndromes, CIII defects are associated with a wide range of clinical presentations, the only common feature being the reduced ubiquinol:cytochrome c oxidoreductase enzymatic activity measured in samples from the subjects under study. The defective factor responsible for CIII malfunction and thus, the molecular pathogenic mechanisms are also widely variable.

Complex III or cytochrome bc1 complex forms the central part of the mitochondrial respiratory chain, oxidizing coenzyme Q and reducing cytochrome c while pumping protons from the matrix to the intermembrane space through the so-called Q-cycle mechanism (Crofts et al., 2008). Mammalian CIII is a multiheteromeric enzyme composed of eleven different subunits (Schagger et al., 1986), one encoded by mitochondrial DNA (mtDNA) and ten by nuclear genes. These eleven subunits constitute the monomeric module of a symmetric dimer (CIII_2_), which constitutes the functionally active form of the enzyme. The complex is embedded in the mitochondrial inner membrane, spanning from the matrix to the inter-membrane space. The crystal structure (Xia et al., 1997; Iwata et al., 1998) differs from the ones of yeast (Hunte et al., 2000) and chicken (Zhang et al., 1998) only because it contains one additional subunit. This extra subunit (Subunit 9) is just the mitochondrial targeting sequence peptide of the Rieske Fe-S protein, which is incorporated into the complex after its cleavage during import into the organelle (Brandt et al., 1993). Three of the 11 subunits contain the catalytic centers: cytochrome b (MT-CYB), cytochrome c1 (CYC1) and the Rieske protein (UQCRFS1). Cytochrome b contains two heme moieties, the low potential (b_L_) and the high potential (b_H_) heme b; CYC1 binds a c-type heme group and the Rieske iron-sulfur protein contains a 2Fe-2S cluster. The exact function of the other eight supernumerary subunits (UQCRC1, UQCRC2, UQCRH, UQCRB, UQCRQ, Subunit 9, UQCR10 and UQCR11) remains to be established (Xia et al., 2013).

The proteins needed for transcription and translation of MT-CYB, as well as CIII assembly factors, are all encoded by nuclear genes.

**FIGURE 1:**
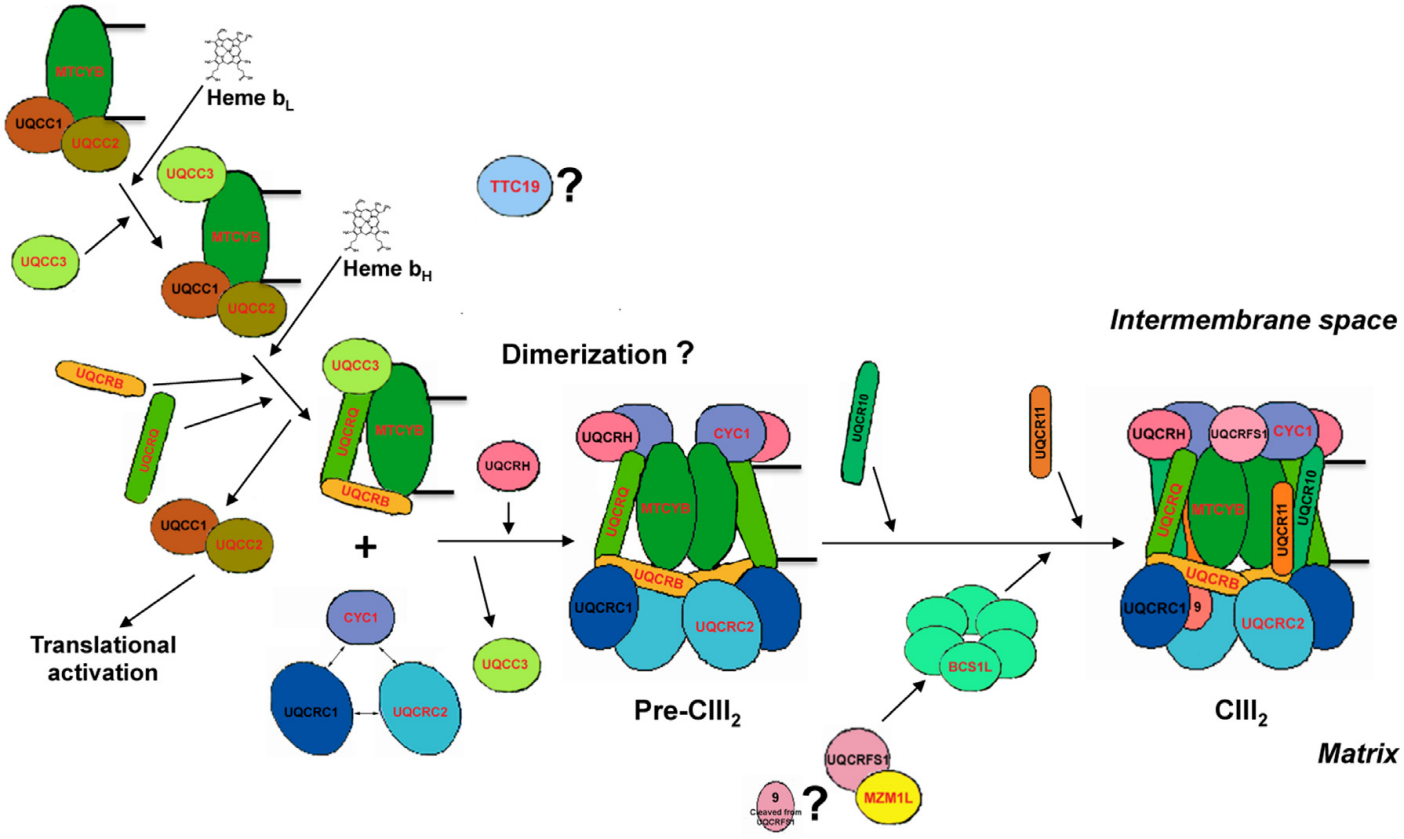
**Human complex III assembly model**. The model is constructed by homology with the available data for *S. cerevisiae* (Zara et al., 2007, 2009a,b; Atkinson et al., 2011; Gruschke et al., 2011, 2012; Wagener et al., 2011; Cui et al., 2012; Smith et al., 2012; Hildenbeutel et al., 2014). The names of the proteins mutated in CIII-deficiency cases are in red. First, MT-CYB is translated in the mitochondrial ribosomes to which UQCC1 and UQCC2 are bound to the exit tunnel, activating its synthesis. The UQCC1-UQCC2 complex remains bound to MT-CYB once it is completely synthesized and incorporated to the mitochondrial inner membrane. Once low-potential heme b (b_L_) is inserted into the catalytic center of cytochrome b, UQCC3 binds to it and then the high potential heme b (b_H_) is incorporated. The UQCC1-UQCC2 dimer is released when UQCRB and UQCRQ bind to MT-CYB, to form the early-stage CIII intermediary, so that UQCC1-UQCC2 dimer becomes available to activate translation of new MT-CYB by binding to the mitoribosome. This system provides a way of regulating MT-CYB translation related to its assembly into CIII. After the formation of the early intermediate MT-CYB+UQCRB+UQCRQ, additional subunits, i.e., UQCRC1, UQCRC2, and CYC1 are incorporated, followed by UQCRH and later UQCR10, to form pre-complex III (pre-CIII_2_). At this point, the complex is already dimeric, but the precise stage at which dimerization occurs is currently unknown. Finally, UQCRFS1 is translocated from the matrix into the inner mitochondrial membrane and is incorporated into pre-CIII_2_. In the matrix, UQCRFS1 is bound and stabilized by MZM1L. Finally the last subunit (UQCR11) joins the nascent complex, so that assembly is completed. TTC19 is necessary for the correct biogenesis of CIII_2_ in human mitochondria, but the step in which it intervenes is not known yet.

Cytochrome bc1 assembly has been mainly studied in the yeast *Saccharomyces cerevisiae.* Yeast deletion mutants have proved to be an extremely useful tool to discover proteins involved in CIII and CIV biogenesis. By using yeast as a model organism, a great deal of CIV assembly factors have been discovered during the last two decades, and the assembly of this enzyme is known in mechanistic detail (Mick et al., 2011; Soto et al., 2012). Although investigation on CIII biogenesis has been far less assiduous, models of CIII assembly in yeast have been developed in the last years (Zara et al., 2004, 2007, 2009b; Gruschke et al., 2011, 2012; Hildenbeutel et al., 2014). According to these models, the different structural subunits start gathering inside several subcomplexes that afterward join to form a pre-CIII. The complex finally matures and becomes active with the addition of the Rieske protein, which is the second to last subunit to be incorporated. A complete understanding on how this incorporation occurs has already been achieved in yeast (Wagener et al., 2011). Thus, in addition to the structural subunits, another set of proteins are involved in the process acting as chaperones, which bind individual subunits or assembly intermediates to stabilize them, or as assembly factors, who act to incorporate subunits during the pathway (Smith et al., 2012). CIII assembly in humans is deemed to be similar to that in yeast (**Figure 1**), given the structural similarity between yeast and mammalian CIII and because several orthologs of yeast assembly factors have been identified in humans as well (Petruzzella et al., 1998; Sanchez et al., 2013; Tucker et al., 2013; Wanschers et al., 2014). However, mechanistic details have been experimentally proven only for a few steps of human CIII assembly. The initial step is the release of MT-CYB from the mitoribosome and its insertion into the mitochondrial inner membrane, a process that requires two assembly factors, UQCC1 and UQCC2 (Tucker et al., 2013). A much later step has also been defined in humans, consisting in the incorporation of the Rieske protein (UQCRFS1), operated by the assembly factor, BCS1L, into a nearly complete dimeric but inactive pre-complex III (pre-CIII_2_; Fernandez-Vizarra et al., 2007; Hinson et al., 2007; Gil-Borlado et al., 2009). The exact mechanism has not been studied in detailed in human systems, but the evidences point to a very similar process as the one in yeast mitochondria. However, there is still very limited information about the intermediate steps, i.e., how the pre-CIII_2_ stage is achieved.

Until 2008, mutations in only three genes were known to be associated with CIII deficiency (Benit et al., 2009; Fernandez-Vizarra et al., 2009). The first to be detected were in *MT-CYB* (OMIM 516020), initially described in the late 1990’s (Andreu et al., 1998, 1999a,b, 2000; De Coo et al., 1999; Keightley et al., 2000). These were usually sporadic mutations causing myopathy and exercise intolerance. Then, mutations in the nuclear gene *BCS1L* were found in CIII-deficient patients (de Lonlay et al., 2001). Defective BCS1L is still the most frequent cause of CIII-defective mitochondrial disease, as more than 25 different pathological mutations associated to very variable clinical presentations have been described (**Table 3**). Finally, a mutation in the *UQCRB* gene was found in a subject with hepatopathy and CIII deficiency (Haut et al., 2003). Since then, the introduction of next generation sequencing (NGS) techniques and the identification of additional assembly factors in yeast (Cruciat et al., 1999; Atkinson et al., 2010; Gruschke et al., 2011, 2012; Mathieu et al., 2011; Cui et al., 2012) corresponding to orthologs in humans, has led to the identification of seven more CIII-disease genes. Three encode CIII structural subunits: *UQCRQ* (Barel et al., 2008), *UQCRC2* (Miyake et al., 2013), *CYC1* (Gaignard et al., 2013) whereas the remaining four encode assembly factors: *TTC19* (Ghezzi et al., 2011)*, LYRM7* (Invernizzi et al., 2013), *UQCC2* (Tucker et al., 2013) and *UQCC3* (Wanschers et al., 2014). All of the mutations in nuclear genes follow an autosomal recessive pattern of inheritance.

This review will describe in detail each of the nuclear-encoded proteins in which mutations have been found to be associated to CIII deficiency (summarized in **Table 1**) in relation to their function and the clinical presentations of the patients suffering from this condition. In an attempt to explain the molecular pathological mechanisms found in the patients we will relate them to the role of the specific protein within the assembly process, bearing in mind that most of the information was obtained in the *S. cerevisiae* system.

**Table 1:** Summary of the proteins encoded in the nuclear genome in which mutations have been associated to Mitochondrial Complex III Deficiency.

Protein	Yeast ortholog	Molecular role	OMIMnumber
**Structural subunits**
UQCRB	Qcr7	Supernumerary subunit	191330
UQCRQ	Qcr8	Supernumerary subunit	612080
UQCRC2	Qcr2 (Cor2)	Supernumerary subunit	191329
CYC1	Cyt1	Catalytic subunit	123980
**Accessory factors**
TTC19	–	Unknown	613814
BCS1L	Bcs1	UQCRFS1 translocase	603647
LYRM7/MZM1L	Mzm1	UQCRFS1chaperone	615831
UQCC2	Cbp6	MT-CYB translational activator and chaperone	614461
UQCC3	Cbp4	MT-CYB chaperone	–

## Structural Subunits

### UQCRB

The daughter of healthy consanguineous Turkish parents is the only known case of CIII deficiency caused by a mutation in the *UQCRB* gene, located on chromosome 8q22 (OMIM 191330). *UQCRB* encodes the human ubiquinone-binding protein of CIII (QP-C subunit or subunit VI; Haut et al., 2003). The girl was born and developed normally until 8 months of age when she presented with acute gastroenteritis. She showed liver enlargement and blood tests revealed mildly elevated hepatic enzymes, hypoglycemia, metabolic acidosis due to high blood lactate levels that increased during metabolic crises. Isolated CIII deficiency was detected in lymphocytes, skin fibroblasts and in a liver biopsy. Furthermore, the amount of cytochrome b was greatly reduced in mitochondria isolated from fibroblasts. The child was reported to have undergone good clinical recovery, with neither psychomotor nor other neurological impairment. Genetic analyses showed a homozygous 4-bp deletion (nucleotides 338–341) in exon 4 of the UQCRB gene, which predicts the synthesis of an abnormal amino acid stretch at the C-terminal end in a highly conserved portion of the protein. Both parents were heterozygous for the same mutation, which was absent in 55 control individuals.

UQCRB is the homolog of the yeast Qcr7 subunit, which is located in the matrix-inner mitochondrial membrane interphase and is deemed to interact with cytochrome b in early stages of the assembly pathway (Zara et al., 2009a; **Figure 1**). Qcr7 incorporation is essential to stabilize hemylated cytochrome b (Hildenbeutel et al., 2014). By analogy, UQCRB must be important for MT-CYB stability, which would explain the low amounts of b-type cytochrome found in the patient mitochondria.

### UQCRQ

*UQCRQ* is a nuclear gene located on chromosome 5q31, which encodes a 9.5 kDa structural subunit of CIII, UQCRQ, also termed subunit VII (OMIM 612080). A deleterious, autosomal recessive mutation consisting of a c.208C > T transition in exon 2 of *UQCRQ*, determining a p.Ser45Phe amino acid change, was found in 25 affected members of a large consanguineous Israeli Bedouin kindred (Barel et al., 2008). All the affected individuals appeared normal at birth but showed delayed development during the first months of life, followed by the development of severe Leigh-like syndrome with profound mental and extrapyramidal signs. Reduced CIII activity was found in skeletal muscle of the probands. Albeit severe and early-onset, the clinical course was progressive, with some individuals still being alive in their thirties.

UQCRQ is embedded in the mitochondrial inner membrane, in close contact to MT-CYB deep in the CIII backbone structure (Iwata et al., 1998). Its yeast homolog, Qcr8, was proposed to form the early cytochrome b subcomplex together with Qcr7 (Zara et al., 2009a; **Figure 1**).

### UQCRC2

Linkage analysis and whole-exome sequencing allowed the identification of a pathological mutation in the *UQCRC2* gene, located on chromosome 16p2, encoding the CIII Core 2 protein (also termed subunit II; OMIM 191329). The same homozygous nucleotide mutation, (c.547C>T) leading to a p.Arg183Trp amino acid change, was found in three members of a Mexican consanguineous family (Miyake et al., 2013). Right after birth, the children presented recurrent episodes of metabolic decompensation with lactic acidosis, hypoglycemia, hyperammonemia, and ketosis but no neurological impairment or severe developmental delay. In fact, the clinical presentation resembled that of the already reported UQCRB mutation (see above). The p.Arg183Trp predicts to disrupt the protein structure at its hydrophobic core, thus affecting CIII stability. In agreement with this prediction, the patient fibroblasts showed a twofold reduction of CIII activity and amount.

According to the CIII structure, Core 2 is facing the matrix in close interaction with core 1 (UQCRC1). In the most recent assembly model of the yeast bc1 complex, the core 2 subunit (Qcr2) is proposed to interact with CYC1 to form one of the assembly intermediates (Zara et al., 2009a; **Figure 1**).

### Cytochrome c1

Mutations in one of the nuclear-encoded catalytic subunits, CYC1 were proved to cause CIII deficiency in two unrelated patients with similar clinical course (Gaignard et al., 2013). The protein is a product of the *CYC1* gene, located on chromosome 8q24 (OMIM 123980). The son of first cousins of Lebanese origin was found to carry a homozygous c.288G>T transversion, by whole-exome sequencing. The consequence of this change is the substitution of an invariant tryptophan residue in position 96 of the protein (p.Trp96Cys). The second patient, a Sri Lankan girl also born to consanguineous parents, carried another missense mutation in both alleles (c.643C>T) which produces a p.Leu215Phe amino acid change. Sequence alignment of CYC1 from different species proved the total conservation of this residue throughout evolution. The main clinical feature in both patients was the recurrent episodes of metabolic failure, usually after minor infections, with hypo or hyperglycemia that was responsive to insulin treatment, and severe hyperlactatemia. In both, neurodegenerative lesions were detected by brain MRI whereas psychomotor regression during metabolic crises was followed by complete recovery and overall normal development.

Immunoblot analyses of fibroblasts and skeletal muscle from both patients showed low steady state levels of CYC1 and a CIII assembly defect. Furthermore, both mutations were predicted to disturb the protein structure, but showed to generate hypomorphic alleles since high copies of the mutant protein could rescue the respiratory defect either in a deletion yeast strain or in the patient cells.

Within CIII, CYC1 is anchored to the inner mitochondrial membrane, and its C-terminus, containing the heme moiety, faces the inter-membrane space. CYC1 receives the electrons from UQCRFS1 to then transfer them to the mobile electron carrier cytochrome c (Iwata et al., 1998). In different yeast strains carrying deletions in structural subunits in which bc1 assembly is arrested, it was shown that CYC1 interacts with Core 1 and Core 2 (Zara et al., 2007). This led the Authors to conclude that the three subunits interact with each other early during CIII assembly (Zara et al., 2009a; **Figure 1**). Considering the similarities in CIII structure and assembly, this would explain the low UQCRC1 and UQCRC2 levels found in the CYC1 mutated fibroblasts and skeletal muscle (Gaignard et al., 2013).

## Accessory Proteins: Assembly Factors

### TTC19

*TTC19* (OMIM 613814), located on chromosome 17p12, encodes a protein shown to be involved in CIII biogenesis. The tetratricopeptide repeat domain-containing protein 19 (TTC19) is present in animals but absent in plants or yeast (Ghezzi et al., 2011). The first cases of CIII deficiency associated to *TTC19* mutations were described in three patients from two unrelated Italian families with early-onset but slowly progressive encephalopathy, and in a fourth patient with late-onset but rapidly progressive neurological failure (Ghezzi et al., 2011). Since the first cases were reported, other *TTC19* mutations have been published always associated with isolated CIII but with different clinical presentations. A progressive neurodegenerative disorder showing severe psychiatric signs and cerebellar disease was found in four Portuguese siblings born to consanguineous parents (Nogueira et al., 2013), Leigh syndrome was reported in a Hispanic child (Atwal, 2013) and cerebellar ataxia in Japanese adult individuals (Morino et al., 2014; Kunii et al., 2015). All the described cases (**Table 2**) carried non-sense or frameshift mutations leading to a truncated protein and, at least in the first reported patient samples, to undetectable TTC19 levels (Ghezzi et al., 2011). However, the clinical output may vary in age of onset, severity, and presence of psychiatric symptoms.

**Table 2:** Summary of the clinical presentations and mutations in TTC19.

Phenotype	TTC19 mutation	Reported in
**Early onset**
Slowly progressive cognitive impairment and ataxia	p.Leu219Ter	Ghezzi et al. (2011)
Slowly progressive developmental delay and language regression, Leigh syndrome	p.Trp186Ter/p.Gly322MetfsTer8	Atwal (2013)
Slowly progressive unsteady gait, learning difficulties, and behavioral alterations	p.Gln77ArgfsTer30	Melchionda et al. (2014)
**Late onset**
Rapidly progressive neurological and psychiatric symptoms	p.Gln173Ter	Ghezzi et al. (2011)
Rapidly progressive psychiatric symptoms, ataxia and pyramidal signs	p.Ala321fsTer8	Nogueira et al. (2013)
Rapidly progressive spinocerebellar ataxia and cognitive impairment	p.Gln277Ter	Morino et al. (2014)
Cerebellar ataxia, spastic paraparesis, loss of deep sensation, mild frontal lobe dysfunction and transient psychiatric symptoms	p.Pro54AlafsTer48	Kunii et al. (2015)

TTC19 was shown to co-immunoprecipitate and co-migrate in Blue-Native Gel Electrophoresis (BNGE) with several CIII structural subunits, suggesting physical interaction. Although its exact function is currently unknown, a role as a chaperone in the first steps of CIII assembly is proposed for this protein, because a proportion of unassembled UQCRC1 and UQCRC2 were found in mutant muscle samples (Ghezzi et al., 2011). In addition, a Ttc19-null *Drosophila melanogaster* model showed profound CIII deficiency associated to neurological impairment in adult individuals but, surprisingly, normal CIII activity in larvae, suggesting developmental control of CIII assembly in flies (Ghezzi et al., 2011).

### BCS1L

Mutations in *BCS1L* on chromosome 2q35 (OMIM 603647) are still the most frequent cause of mitochondrial CIII isolated deficiency. BCS1L is a member of the AAA+ (ATPases associated with diverse cellular activities) family of proteins. Yeast Bcs1 or human BCS1L are needed for the final steps of bc1 complex assembly, where the Rieske Fe-S protein (Rip1 or UQCRFS1) and the smallest subunit (Qcr10 or UQCR11) are incorporated into the pre-CIII_2_ to complete the process (Nobrega et al., 1992; Cruciat et al., 1999; Fernandez-Vizarra et al., 2007; Wagener et al., 2011). Bcs1 is not exactly a chaperone, as it was demonstrated to act as a translocase of the Rieske Fe-S protein (Rip1), moving it from the matrix to the inner mitochondrial membrane through interaction with the Rip1 N-terminal domain and ATP hydrolysis (Wagener et al., 2011; Cui et al., 2012). Bcs1 ATPase activity seems to be necessary not only for its function but also to couple the mitochondrial energy state to CIII biogenesis (Ostojic et al., 2013).

**FIGURE 2:**
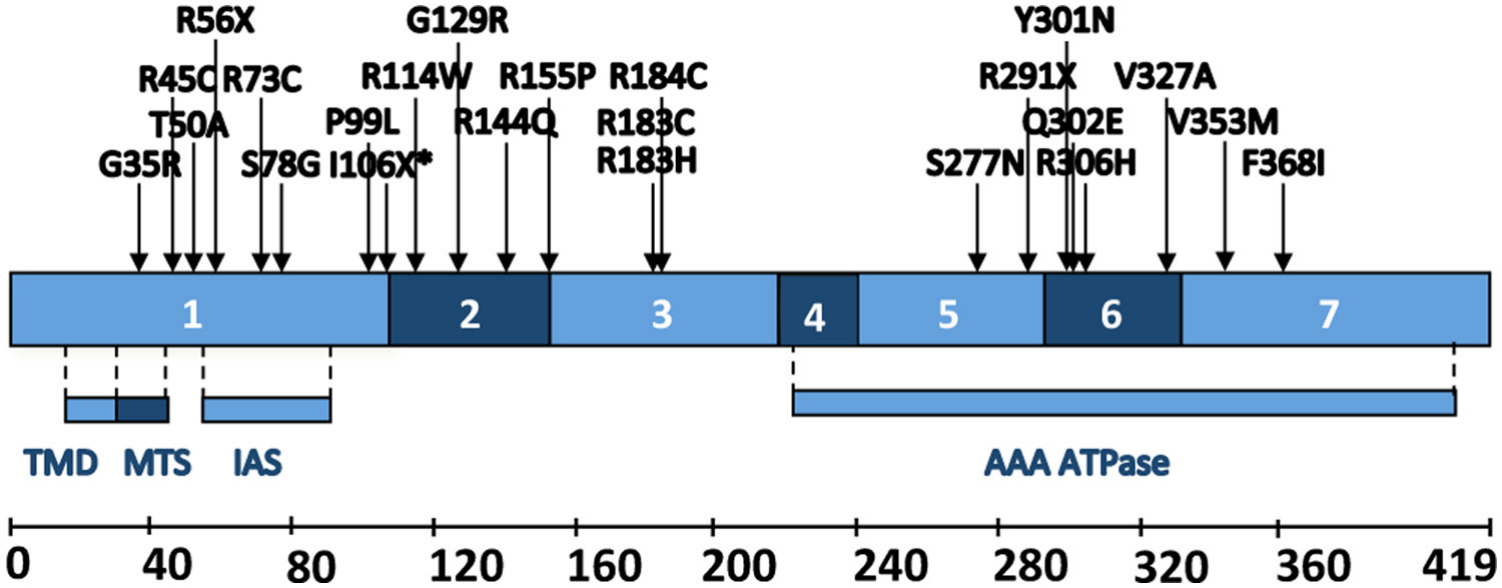
**Schematic representation of human BCS1L**. Different coding exons and functional regions of the protein. TMD, trans-membrane domain; MTS: mitochondrial targeting sequence; IAS: import auxiliary sequence. Positions and amino acid changes of the pathological mutations described up to date are also depicted.

BCS1L mutations (**Figure 2**) are associated with a wide variety of clinical manifestations with different tissue involvement and disease progression (**Table 3**), ranging from multivisceral GRACILE syndrome (growth retardation, aminoaciduria, cholestasis, iron overload, lactic acidosis, and early death; OMIM 603358; Visapaa et al., 2002; Lynn et al., 2012; Kasapkara et al., 2014); to congenital metabolic acidosis, neonatal proximal tubulopathy and/or liver failure with or without encephalopathy (de Lonlay et al., 2001; De Meirleir et al., 2003; Blazquez et al., 2009; Gil-Borlado et al., 2009; Ramos-Arroyo et al., 2009; Ezgu et al., 2013); to isolated severe mitochondrial encephalopathy (Fernandez-Vizarra et al., 2007); to milder phenotypes such as Björnstad syndrome (sensorineural hearing loss and pili torti; OMIM 262000; Hinson et al., 2007; Siddiqi et al., 2013), a neurological syndrome with long-term survival (Tuppen et al., 2010) or neuro-psychiatric manifestations (Al-Owain et al., 2013). In contrast to the other BCS1L mutations, no CIII deficiency was originally reported for the GRACILE mutation, leading to p.Ser78Gly change which is part of the Finnish heritage disease repertoire (Fellman, 2002). However, a more recent report demonstrated decreased CIII amount and activity in liver, kidney and heart of a GRACILE mutant patient (Kotarsky et al., 2010). The other BCS1L mutations are indeed all associated with CIII deficiency, either isolated or in combination with reduced CIV and sometimes CI activities, with reduced amount of UQCRFS1 and with accumulation of unstable pre-CIII_2_ (Fernandez-Vizarra et al., 2007; Hinson et al., 2007; Moran et al., 2010).

**Table 3:** Summary of the clinical presentations and mutations in BCS1L.

	Phenotype	BCS1L mutation	Reported in
**Purely Visceral**	GRACILE syndrome	p.Ser78Glyp. Pro99Leu	Visapaa et al. (2002)Kasapkara et al. (2014)
	Complex III deficiency, lactic acidosis, hepatopathy, developmental delay, sensorineural hearing loss	p.Thr50Ala	Blazquez et al. (2009)
	GRACILE + CIII deficiency + neurological symptoms	p.Arg56Ter/p.Val327Ala Intron 1 -588T>A/Intron 2 321G>T p.Ser78Gly/p.Arg144Gl np.Arg56Ter/ Intron 1 -588T>A^∗^	Visapaa et al. (2002)Lynn et al. (2012)
	Complex III deficiency, tubulopathy, encephalopathy and liver failure	p.Ser277Asn p.Pro99Leu p.Arg155Pro/p.Val353Met p.Arg45Cys/p.Arg56Ter g.1181A>G+g.1164C>G/p.Arg56Ter^∗^	de Lonlay et al. (2001)De Meirleir et al. (2003)Gil-Borlado et al. (2009)Ramos-Arroyo et al. (2009)
**Pure Encephalopathy**	Complex III deficiency, encephalopathy, muscle hypotonia, psychomotor delay, pili torti	p.Gly35Arg/p.Arg184Cys p.Arg73Cys/p.Phe368Ile p.Arg183Cys/p.Arg184Cys	Hinson et al. (2007)Fernandez-Vizarra et al. (2007)
**Milder Phenotypes**	Björnstad syndrome	p.Arg183His p.Ile106Ter (IVS2+1G>T)/p.Arg306His p.Arg306His/p.Arg114Trp p.Arg291Ter/p.Gln302Glu p.Tyr301Asn	Hinson et al. (2007)Siddiqi et al. (2013)
	Muscle weakness, focal motor seizures, optic atrophy, long-survival	p.Gly129Arg	Tuppen et al. (2010)
	Behavioral alterations, hypomania/psychosis	p.Gly129Arg	Al-Owain et al. (2013)

The BCS1L protein contains several functional domains (**Figure 2**) necessary for its activity and stability (Folsch et al., 1996; Cruciat et al., 1999; Nouet et al., 2009): a transmembrane helix, a mitochondrial signal peptide plus an import auxiliary sequence, the AAA-domain and a Bcs1-specific sequence. Known pathological mutations are scattered throughout the different regions of the BCS1L sequence, with the exception of the transmembrane domain (**Figure 2**). In addition, some mutations have also been found in splice sites and in the 5′-UTR of the mature *BCS1L* mRNA (**Table 3**). It is difficult to relate the position of the mutation to the pathological consequences at the biochemical and clinical levels. Most authors hypothesize that the clinical variability shown by the BCS1L mutated patients must be due to the nature of the amino acid substitution and its differential expression in different tissues (Hinson et al., 2007; Kotarsky et al., 2007; Ramos-Arroyo et al., 2009). Different *BCS1L* mutations, investigated in six patient-derived skin cultured fibroblasts, were found to impair a number of different parameters, such as CIII activity and assembly, import of the protein into mitochondria, supramolecular associations of BCS1L, cell growth, shape of the mitochondrial network, ROS levels and antioxidant defenses, and apoptosis (Moran et al., 2010). Interestingly, the extent of the functional alterations in cultured cells correlated with the severity of the clinical presentation.

A consistent hallmark of GRACILE syndrome is liver iron overload, leading to the proposal of a role for BCS1L in cellular iron homeostasis (Visapaa et al., 2002). However, iron overload is present in some non-GRACILE cases and absent in others (Ramos-Arroyo et al., 2009; Moran et al., 2010). To better characterize the pathophysiological consequences of the GRACILE mutation, a knock-in mouse has been created, being the only viable animal model of CIII deficiency reported to date (Leveen et al., 2011). Homozygous mutant mice show growth retardation starting at 3 weeks and eventually die before 6 weeks from birth. They show progressive hepatopathy and profound CIII deficiency in liver, and to a lesser degree in heart and kidney as well. Interestingly, whilst the levels of Bcs1l in homozygous mutant liver and kidney were very low at any age, the amount of Uqcrfs1 incorporated into CIII_2_ progressively decreased over time, becoming almost undetectable just before death.

### MZM1L (LYRM7)

Human *LYRM7*, located on chromosome 5q23.3 (OMIM 615831), encodes the LYR (leucine/tyrosine/arginine)-motif protein 7, a member of the Complex1_LYR-like superfamily (Angerer, 2013). LYR motifs are the molecular signature of proteins that contain or assist in the delivery of Fe-S clusters (Maio et al., 2014). LYRM7 is highly homologous to the yeast protein Mzm1, which was originally described as necessary to maintain the mitochondrial pool of zinc (Atkinson et al., 2010), but was later demonstrated to be an assembly factor specific to the bc1 complex (Atkinson et al., 2011; Cui et al., 2012). The human protein can functionally complement the respiratory defect of a Mzm1-deleted yeast strain (Sanchez et al., 2013). Because of the similarities between both proteins, we proposed MZM1L as an alternative name for LYRM7 (Sanchez et al., 2013).

As already mentioned, the last step in CIII assembly involves the incorporation of Rip1/UQCRFS1 protein into CIII in the inner mitochondrial membrane after it has been imported into the matrix, processed to a mature form and endowed with a 2Fe-2S cluster cofactor (Hartl et al., 1986). The yeast and human proteins work as a stabilizing chaperone of the Rieske protein in the mitochondrial matrix, interacting with its C-terminal domain and holding it there prior to its translocation to the inner mitochondrial membrane and incorporation to CIII (Cui et al., 2012; Sanchez et al., 2013). A possible role for LYRM7 in Fe-S cluster delivery is also suggested by its interaction with the HSC20 cochaperone (Maio et al., 2014).

Genetic screening of *LYRM7* in unresolved cases of CIII deficiency, led to the identification of a homozygous c.73G>A transition, which predicts a p.Asp25Asn amino acid change. Yeast studies confirmed the pathogenicity of the mutation (Invernizzi et al., 2013). The affected child was born to Moroccan healthy first cousins. Her development was normal until 20 months of age, when she presented progressive weakness with anemia associated with low plasma iron. A month later, during a febrile infection, she developed severe encephalopathy and metabolic acidosis. Severe psychomotor regression persisted until 28 months of age, when she died of respiratory failure.

**FIGURE 3:**
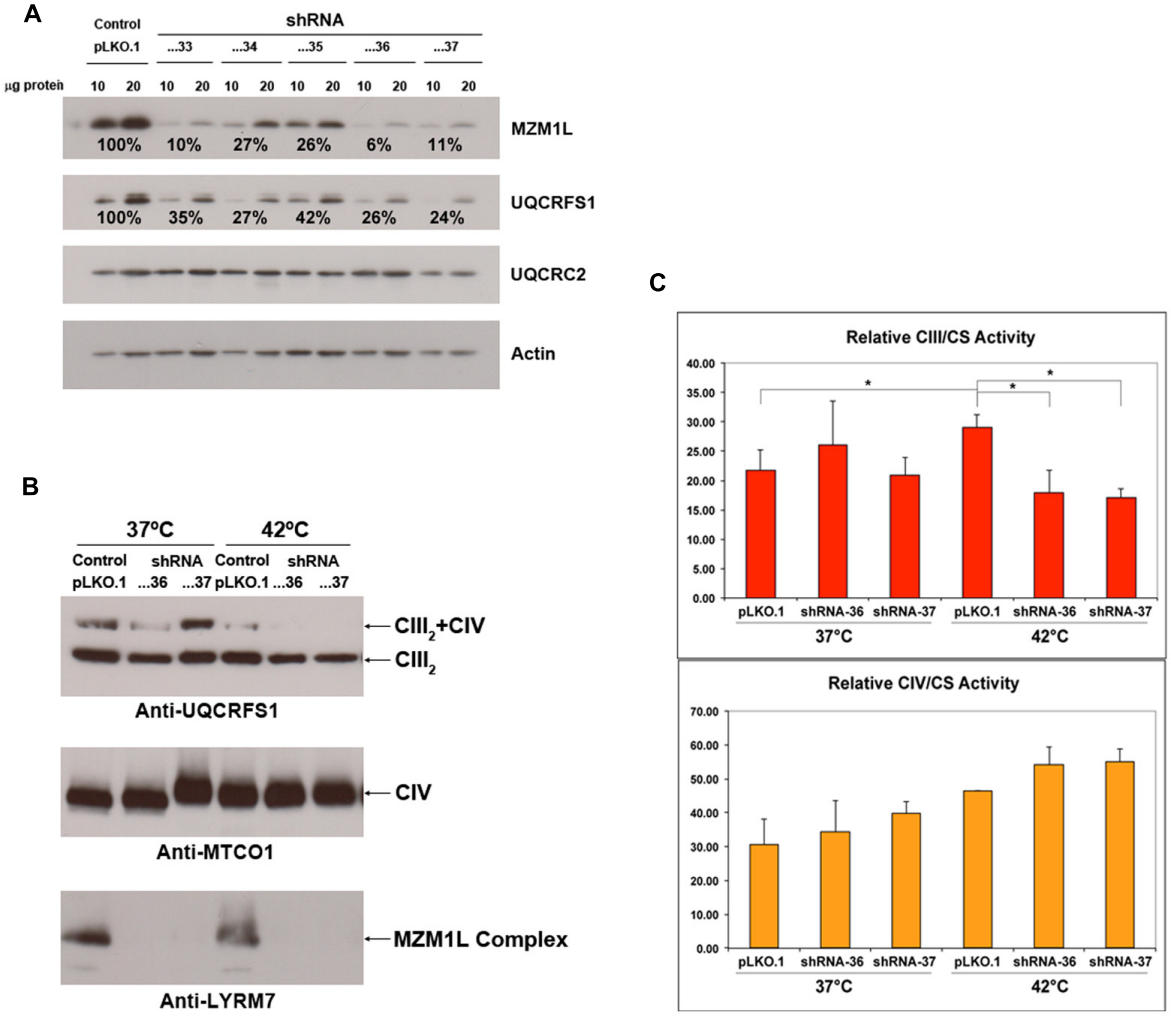
**MZM1L RNA interference experiments in HeLa cells**. Five different shRNA sequences cloned in the pLKO.1 lentiviral vector (TRCN0000064433-37), purchased from the MISSION® shRNA Library (Sigma-Aldrich) were stably transduced. **(A)** The steady state levels of the indicated proteins were quantified by SDS-PAGE, Western blot and immunodetection with specific antibodies: anti-LYRM7 (MZM1L) and Actin (Sigma-Aldrich) and UQCRFS1 and UQCRC2 (Mitosciences-Abcam). Actin was used as the normalizer/loading control. The shRNA sequences...33, …36, and …37 were the most efficient in knocking down MZM1L expression. **(B)** Blue-Native PAGE, Western blot and immunodetection analysis of knocked down and control digitonin-treated cells (Nijtmans et al., 2002) grown either at 37^∘^C or at 42^∘^C for 24 h. Anti-MTCO1 was from Mitosciences-Abcam. **(C)** Spectrophotometric CIII and CIV enzyme activities of the RNAi and control cells grown either at 37^∘^C or at 42^∘^C for 24 h. ^∗^*p* < 0.05 according to the ANOVA *Post-Hoc* LSD test (SPSS 16.0 software for Windows).

To further characterize the molecular function of MZM1L, we performed RNAi experiments in HeLa cells (**Figure 3A**). Similar to the Mzm1 deletion yeast strain (Atkinson et al., 2011), low levels of MZM1L caused a decrease in the total amount of UQCRFS1 (**Figure 3A**), while UQCRC2 was unaffected. However, in normal culture conditions, the amount of UQCRFS1 incorporated into CIII_2_ and the CIII enzyme activity was the same as in control cells (**Figures 3B,C**). Only by exposing the interfered cells to heat stress (incubation at 42^∘^C for 24 h) was the reduced CIII assembly and activity manifested (**Figures 3B,C**). Thus, the absence of MZM1L makes UQCRFS1, unstable but only the fraction residing in the matrix that has not been assembled into the complex. These results support the idea that MZM1L is necessary to stabilize UQCRFS1 in the matrix but is not involved in its translocation, which is virtually normal even when MZM1L is absent. Similar to Mzm1-deficient yeast (Atkinson et al., 2011), the phenotype is thermo-sensitive, probably because at high temperatures the unassembled Rieske protein is more prone to degradation and/or aggregation (Cui et al., 2012). This could explain why the clinical course drastically worsened in the LYRM7 mutated patient after a febrile episode (Invernizzi et al., 2013).

### UQCC2

Sequencing the “MitoExome,” i.e., through the specific capture of both mtDNA and ∼1,000 nuclear genes encoding (part of) the mitochondrial proteome (Calvo et al., 2012), a mutation in *UQCC2* (*C6orf125*; OMIM 614461) was discovered in a patient with profound CIII deficiency (Tucker et al., 2013). This subject also presented secondary reduction in CI and CIV activities. The mutation, found in homozygosis in exon 2 (c.214-3C>G), caused altered splicing of the transcript and the absence of the protein (Tucker et al., 2013). The consequence for the patient, a boy born to consanguineous Lebanese parents, was severe metabolic acidosis documented 12 h after birth. He also had dysmorphic features, delayed neurological development and sensorineural hearing impairment, and later developed autistic features and aggressive behavior. He was alive at 9 years of age when clinical follow-up was lost.

**FIGURE 4:**
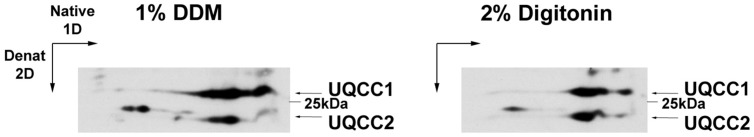
**Co-migration of human UQCC1 and UQCC2 in Blue-Native PAGE**. Mitoplasts from HeLa cells were solubilized using either 1% dodecylmaltoside (DDM) or 2% digitonin and then run through a NativePAGE^TM^ 3–12% native gel (Life Technologies). After the lanes were excised, treated with denaturing solution and run through a NuPAGE^TM^ 4–12% Bis-Tris denaturing gel (Life Technologies). The gels were then transferred to PVDF membranes and immunoblotted with antibodies recognizing UQCC1 (UQCC) and UQCC2 (MNF1) both from Abcam.

UQCC2 is the ortholog of yeast Cbp6, which interacts with Cbp3, homolog to human UQCC1. UQCC2 and UQCC1 were also shown to interact, because the presence of one is required for the stability of the other (Tucker et al., 2013). The two proteins co-migrate in Blue-Native gels (**Figure 4**), showing a pattern very similar to the yeast proteins (Gruschke et al., 2011, 2012; Hildenbeutel et al., 2014). In yeast mitochondria, the Cbp3–Cbp6 complex binds to the mitochondrial ribosome exit tunnel where it is necessary for the efficient synthesis of cytochrome b and the first steps of bc1 complex assembly (Gruschke et al., 2011, 2012). Furthermore, the Cbp3–Cbp6 complex regulates cytochrome b synthesis linking it to its assembly, because once the translation of cytochrome b is completed, the Cbp3–Cbp6 complex remains bound to the peptide in the mitochondrial inner membrane while another protein, Cbp4, is recruited. Unless other structural subunits (Qcr7 and Qcr8) bind to cytochrome b, the Cbp3–Cbp6 complex is sequestered and cannot bind again to the ribosome to activate the synthesis and release of new cytochrome b (Gruschke et al., 2011, 2012; Hildenbeutel et al., 2014).

Similar to Cbp6-deleted yeast model, UQCC2-deficient cells show greatly reduced MT-CYB synthesis, whereas the amount of CIII is recovered when the wild-type protein is expressed (Tucker et al., 2013). The same report demonstrated the physical interaction between UQCC2 and newly synthesized MT-CYB.

### UQCC3

Bioinformatics analyses predicted that the protein encoded in the *C11orf83/UQCC3* gene might be the ortholog of the yeast protein Cbp4, another cytochrome bc1 complex assembly factor (Wanschers et al., 2014). Mutational screening in a patient showing isolated CIII deficiency in skeletal muscle, revealed the presence of a homozygous c.59T>A mutation producing a p.Val20Glu amino acid change, in a position where there are only hydrophobic residues in other metazoan homologs. Both parents were consanguineous, and heterozygous for the mutation. The patient showed very early-onset symptoms, presenting hypoglycemia and severe lactic acidosis already in the first day of life. She suffers from muscle weakness from birth, with severely delayed psychomotor development. Biochemically, cultured fibroblasts showed very reduced levels of assembled CIII and low MT-CYB synthesis and stability.

Yeast Cbp4 assists the first steps of CIII assembly, binding to the cytochrome b-Cbp3-Cbp6 intermediate once it is released from the mitoribosome, but is not required for cytochrome b translation (Gruschke et al., 2012). These interactions can occur only once the b_L_ site of cytochrome b has been hemylated (Hildenbeutel et al., 2014).

There are strong indications that the mutation found in the reported patient is pathogenic: the type of amino acid change, which makes the protein unstable; its absence in a number of controls; and segregation with the disease (Wanschers et al., 2014). Also, the prediction of UQCC3 as being the Cbp4 ortholog explains the CIII deficiency. However, UQCC3 is shorter than Cpb4 (93 vs. 147 amino acids) and their C-terminal sequences are not homologous. Furthermore, human UQCC3 presents a different topology inside mitochondria and is not able to complement a Cbp4-deficient yeast strain (Wanschers et al., 2014). In addition, knocked-down expression by RNAi in control cells did not affect CIII assembly or activity and the patient’s CIII defect in fibroblasts could not be rescued by expression of exogenous wild-type recombinant UQCC3.

## Concluding Remarks

Although the genetic definition of CIII deficiency has remarkably expanded in the last 5 years, ∼50% of the cases remain genetically unsolved. This is likely due to a still sketchy understanding of the assembly process of CIII and limited knowledge of the specific assembly factors required for this process to be completed. As stated above, the model currently favored for CIII assembly (**Figure 1**) is based on studies in *S. cerevisiae,* but several lines of evidence supports the idea that a very similar process occurs in humans (Fernandez-Vizarra et al., 2007; Sanchez et al., 2013; Tucker et al., 2013). Nevertheless, even in yeast mechanistic understanding has been achieved only for the initial and final steps of the process, i.e., the events just following the *de novo* synthesis of cytochrome b (Gruschke and Ott, 2010; Gruschke et al., 2011, 2012; Hildenbeutel et al., 2014) and the incorporation of the Rieske protein which rapidly leads to assembly completion (Atkinson et al., 2011; Wagener et al., 2011; Cui et al., 2012). Whilst OXPHOS accessory proteins that directly interact with structural subunits seem to be conserved from yeast to humans, other factors, for instance TTC19, do not appear to have yeast orthologs (Ghezzi et al., 2011). This reflects evolutionary divergence as well as the differences in the mitochondrial genetic system and MRC organization between yeast and metazoans. The most obvious dissimilarity is the presence in mammals of the multimeric complex I and its interaction with CIII and CIV, to form the so-called supercomplexes or respirasomes (Acin-Perez et al., 2008; Moreno-Lastres et al., 2012; Lapuente-Brun et al., 2013). The easy genetic manipulation and the metabolic flexibility of *S. cerevisiae*, a facultative fermentative organism, make it an extremely useful model to study OXPHOS biogenesis. By genetic data mining and phylogenetic analysis, yeast factors have been key to discover human orthologs and model in yeast some of the mutations found in patients (Barrientos, 2003; Meunier et al., 2013), including assembly factors and chaperones such as BCS1L (de Lonlay et al., 2001; Visapaa et al., 2002; Fernandez-Vizarra et al., 2007; Nouet et al., 2009; Meunier et al., 2013) and MZM1L (Invernizzi et al., 2013).

A next, formidable challenge will be to unravel the mechanisms and factors specific to mammalian CIII biogenesis. In this respect, the genetic characterization of CIII defective subjects, by NGS technology, and new tools offered by proteomic techniques applied to human cells and animal models will be fundamental to make progress in this field.

## Conflict of Interest Statement

The authors declare that the research was conducted in the absence of any commercial or financial relationships that could be construed as a potential conflict of interest.
